# Reviewing Solutions of Scale for Canine Rabies Elimination in India

**DOI:** 10.3390/tropicalmed5010047

**Published:** 2020-03-23

**Authors:** Andrew D. Gibson, Ryan M. Wallace, Abdul Rahman, Omesh K. Bharti, Shrikrishna Isloor, Frederic Lohr, Luke Gamble, Richard J. Mellanby, Alasdair King, Michael J. Day

**Affiliations:** 1 Mission Rabies, 4 Castle Street, Cranborne, Dorset BH21 5PZ, UK; 2 The Royal (Dick) School of Veterinary Studies and the Roslin Institute, Easter Bush Campus, The University of Edinburgh, Roslin, Midlothian EH25 9RG, UK; Richard.Mellanby@ed.ac.uk; 3 United States Centers for Disease Control and Prevention, Poxvirus and Rabies Branch, Atlanta, GA 30333, USA; 4 Commonwealth Veterinary Association 123, 7th B Main Road, 4th Block West, Jayanagar, Bangalore 560011, Karnataka, India; 5 State Institute of Health and Family Welfare, Parimahal, Kasumpti, Shimla 171009, Himachal Pradesh, India; 6 Bangalore Veterinary College, KVAFSU, Hebbal, Bangalore 560024, Karnataka, India; 7 Merck Animal Health, Madison, NJ 07940, USA; 8 World Small Animal Veterinary Association and School of Veterinary and Life Sciences, Murdoch University, Murdoch 6150, Australia

**Keywords:** rabies, dog, vaccination, campaign, scale-up, polio

## Abstract

Canine rabies elimination can be achieved through mass vaccination of the dog population, as advocated by the WHO, OIE and FAO under the ‘United Against Rabies’ initiative. Many countries in which canine rabies is endemic are exploring methods to access dogs for vaccination, campaign structures and approaches to resource mobilization. Reviewing aspects that fostered success in rabies elimination campaigns elsewhere, as well as examples of largescale resource mobilization, such as that seen in the global initiative to eliminate poliomyelitis, may help to guide the planning of sustainable, scalable methods for mass dog vaccination. Elimination of rabies from the majority of Latin America took over 30 years, with years of operational trial and error before a particular approach gained the broad support of decision makers, governments and funders to enable widespread implementation. The endeavour to eliminate polio now enters its final stages; however, there are many transferrable lessons to adopt from the past 32 years of global scale-up. Additionally, there is a need to support operational research, which explores the practicalities of mass dog vaccination roll-out and what are likely to be feasible solutions at scale. This article reviews the processes that supported the scale-up of these interventions, discusses pragmatic considerations of campaign duration and work-force size and finally provides an examples hypothetical resource requirements for implementing mass dog vaccination at scale in Indian cities, with a view to supporting the planning of pilot campaigns from which expanded efforts can grow.

## 1. Introduction

A century of experience in the field by different actors has demonstrated that the key to achieving canine rabies elimination lies in mass vaccination of dogs which serve as a reservoir and vector population, particularly the proportion of the dog population that is free roaming. Canine rabies kills more people than any other zoonotic disease and the relatively low transmissibility of the virus between dogs makes it a prime candidate for widespread elimination [1,2,3,4]. Decades of research have repeatedly emphasized that canine rabies elimination through vaccination of 70% of the dog population is not only technologically and biologically feasible, but also highly cost-effective [5,6,7,8]. Nevertheless, practical understanding as to how to successfully grow and sustain vaccination campaigns in endemic regions remains challenging and prompts frequent calls to focus research efforts on the technical aspects of vaccine delivery at scale, including consideration of resource mobilization and operational feasibility [9,10,11,12].

India is estimated to carry the greatest burden of rabies globally; however, examples of effective, large scale dog vaccination campaigns are currently lacking [13]. With the national elimination of Polio in 2011, India demonstrated an ability to mobilize and coordinate massive vaccination efforts [14], politically showcasing the capability of disease elimination efforts in South East Asia [15]. The large free roaming dog population and abundance of loosely owned dogs in India presents practical challenges to mass immunization [16,17]; however, if a scalable method for mass dog vaccination were to be demonstrated in India, it is possible that countries in the surrounding region would follow.

A state-wide dog vaccination campaign has been established in Goa state where over 100,000 dogs are vaccinated through door-to-door (DD) and capture-vaccinate-release (CVR) methods on a region-by-region annual cycle. This has resulted in a decline in both human and canine cases (Government of Goa data on human and canine rabies incidence); however, the challenge remains of how to amplify this approach from Goa, India’s smallest state, to larger states across the Indian sub-continent. Reviewing the operational characteristics of successful rabies vaccination campaigns deployed at a similar continental scale may help to identify the inflexible starting points for campaign structure, from which methods can be projected to estimate resource requirement and cost, as well as identifying gaps in existing tools.

Although there are several focal or periodic examples, there are few reports of systematic national dog vaccination campaigns from low and middle income countries sustained over the timeframes required to achieve rabies elimination [18,19,20,21,22,23,24]. Those that are available do not provide insight into vaccination method, resource requirement, cost, canine rabies impact, geographic distribution or vaccination coverage [25]. The rabies elimination campaigns of Latin America were therefore selected for review, as well as operational aspects of the India polio immunization campaign.

Many transferrable lessons can be gleaned from the Global Polio Eradication Initiative, which launched over 30 years ago and now enters its final stages [26]. Reflection on both the successes and criticisms of the early stages of advocacy, campaign planning and resource mobilization may guide ambitions to grow large scale dog vaccination activities [27].

Identifying a feasible and effective method for immunizing dogs on a massive scale is prerequisite to national campaign planning, budgeting and resourcing. As each country has different geographical, social, political and logistical challenges, this is best achieved through local pilot campaigns which generate evidence to guide the methodical expansion of activities. Ultimately, regional and national decision makers will only be convinced by an operational plan that has an established track record of successful implementation and that can be economically and logistically scaled up accordingly. In a country the size of India, rabies control will require a level of engagement and resource mobilisation akin to that seen during efforts to eradicate poliomyelitis and expectations and timelines should be considered in this light.

This article is divided into three sections:

(1) A review of fixed campaign determinants used to evaluate campaign feasibility.
(2) A review of the factors typical to successful massive vaccine distribution interventions using two case studies; immunization of dogs against rabies in Latin America and the Caribbean and mass vaccination of children against polio in India.
(3) Estimation of campaign requirements for a hypothetical metropolitan campaign in India using a campaign planning tool, VaxPLAN [28].

## 2. Fixed Campaign Determinants

High income countries benefit from established, widely distributed human and veterinary healthcare systems, which are able to immunize susceptible individuals as they enter the population, thus maintaining the required herd immunity within the animal and human populations to combat many diseases [29]. In low and middle-income countries, however, sustained and widespread healthcare infrastructure is often not available and so to achieve communicable disease control or elimination in the short-term, intensive periodic provision of vaccine is required to achieve and sustain herd immunity [30,31]. This is generally done through campaigns, whereby a workforce is trained and equipped to deliver vaccine to the susceptible population for a defined period of time.

In the formative stages of campaign development, small scale pilot projects provide an opportunity to develop understanding, experience and expertise in a given approach as well as the necessary infrastructure and training practices to be created. Once an effective method has been established, contraction of the campaign period might be possible. A shorter campaign period is economically and politically preferable as it enables for the concentration of human resource, expertise and public awareness activities; as well as making use of a government labour force and local media at scale.

It is necessary to rapidly identify constraints to campaign structure such as campaign duration, vaccination target and rate of vaccination, which are fixed by the local setting. Once these constraints are defined it is possible to estimate the resource requirements and to evaluate the feasibility, scalability and sustainability of proposed methods. Where the outcome appears infeasible, then it is necessary to reflect on the fixed parameters to identify which can be modified. These fixed parameters are:

### 2.1. Vaccination Target

Within a defined geographic area the size of the susceptible host population will determine the approximate number of vaccine doses that must be delivered to achieve herd immunity. Although early campaigns may purposefully aim to reach a low proportion of the population so that scalable methods can be explored and refined; ultimately, a minimum number of individuals will need to be vaccinated across a given area over a defined time period in order to achieve herd immunity and interrupt endemic circulation of the virus [32,33]. Setting low initial vaccination targets may, however, be counter-productive if early campaigns result in no noticeable impact on rabies incidence in the area.

The removal or culling of dogs as a part of dog vaccination is not recommended and therefore campaigns must be planned around how to access a given dog population for vaccination [23,34].

### 2.2. Duration and Frequency of Vaccination

The duration and frequency of the campaign is integral to all other aspects of campaign planning, including the number of staff required, stock (i.e., vaccine, equipment), logistics, costs and community engagement. Campaign planners must consider two factors when determining the duration and frequency of campaign implementation: the biological rationale and the logistical rationale. 

*Biological rationale*: Maintenance of herd immunity in a dog population is influenced by numerous biological factors, including the rate of population turnover, the duration of immunity achieved by the vaccine, and the health status of the dog population. Ecological modeling studies and field experience have shown that the annual vaccination of over 70% of the dog population can stop transmission and eventually lead to elimination if repeated over several years [32,33,35,36]. Depending on these biological factors in the target population, the ideal duration and frequency of vaccination may differ greatly across populations. Healthy populations of well-cared for dogs that receive a high-quality vaccine may maintain adequate levels of herd immunity for multiple years [37]. Whereas dog populations with high rates of turnover and receiving low-quality vaccines may require vaccination multiple times each year.

*Logistical rationale*: Public health initiatives often suffer from a lack of adequate resources, and realistic expectations of campaign duration need to consider the political, infrastructural and human capacities that are available. When these resource are readily available, coordinated campaigns should be synchronized over a short period of time to maximize resource allocation and efficiency [38]. However, when resources are not readily available, planners may need to compromise for less frequent campaigns which last for longer durations.

Sustained mass dog vaccination at national scale is likely to require the government veterinary and para-veterinary workforce, with additional contribution from NGOs, private veterinarians and volunteers [39]. Each country will have a limited period for which it is possible for government veterinary staff to be seconded to rabies control, whilst the private veterinary sector, NGOs and volunteers are likely to only remain engaged for a finite period of weeks or months.

**Figure 1:**
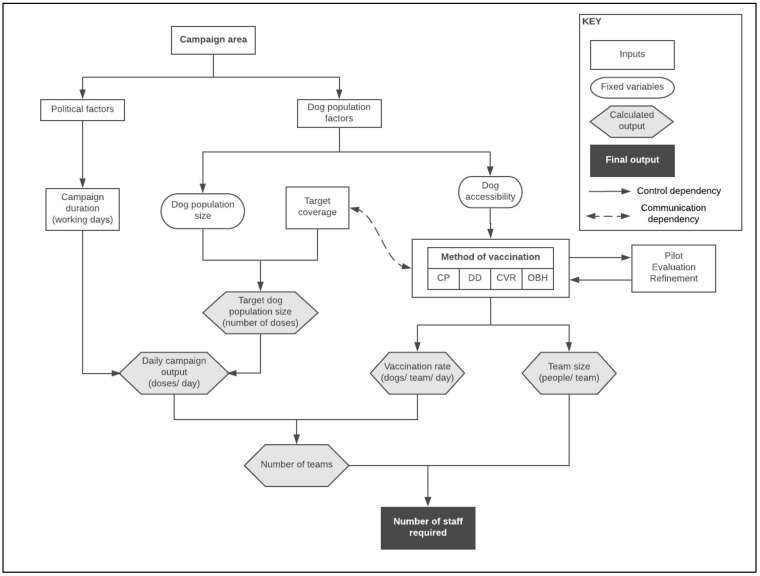
Dependency graph showing the interwoven dependencies involved in vaccination campaign planning.

This maximum campaign duration may change if political support for rabies control increases; however, defining an initial set duration is a useful starting point for campaign planning as it inversely correlates with the number of vaccination teams required and therefore the total number of staff to be recruited (Figure 1) [9].

(1) $$Campaign\;duration\;\left(days\right)=\frac{Target\;number\;of\;vaccinations}{\left(Number\;of\;teams\right)*\left(Vaccinations\;per\;team\;per\;day\right)}$$

### 2.3. Rate of Vaccination

The rate at which dogs can be vaccinated will depend on the method of vaccination, dog ownership practices, dog accessibility, competence of staff and community engagement. For an experienced team, there should be a relatively predictable rate of vaccination (dogs vaccinated per team per day) for a given vaccination method in a specific setting. It may be possible to increase the initial mean rate of vaccination by improving training protocols, adjusting the vaccination method or increasing community involvement; however, once these factors have been optimized, the number of vaccinations delivered per team per day will plateau. The number of teams required to vaccinate a given number of dogs over a defined time period can therefore be estimated. Pilot studies to evaluate rates of vaccination by population-type and method-type are critical to planning efficient campaigns for an early-stage rabies elimination program [28].

Box 1 **Methods of dog vaccination.**

**Central Point (CP)**

Temporary clinics are positioned throughout communities and promoted for people to bring their dogs for vaccination on a particular day. Teams generally consist of a vaccinator, an animal handler and an assistant who sets up and coordinates the clinic for the day. This method relies on a sufficient proportion of the dog population being owned by people who are motivated and able to bring their dogs for vaccination.

**Door to door (DD)**

Vaccination teams travel through communities, visiting households and requesting people to present their dogs for vaccination. Generally, a DD team will consist of one or two people travelling on foot or by motorized bike. Although basic dog handling equipment may be carried, these teams are generally only able to vaccinate accessible dogs that can be easily held for parenteral vaccination.

**Capture vaccinate release (CVR)**

Teams move through communities using nets to catch roaming dogs that cannot be readily handled for vaccination. Teams generally consist of a vaccinator, an assistant, a driver and net catchers. The team structure depends on the local setting; however, generally three to four catchers are needed to successfully catch dogs using the CVR method. In some settings the same teams may also concurrently perform the DD method to vaccinate dogs that can be held for vaccination (CVR-DD); however, this does not make efficient use of the net catching resource whilst compliant dogs are being restrained by hand. Therefore, in settings where CVR is used, it is recommended that separate DD and CVR teams work in parallel through the region to efficiently vaccinate both accessible and inaccessible dogs.

**Oral bait handout (OBH)**

Vaccination teams distribute baits containing ORV to dogs which cannot be held for parenteral vaccination. Dogs offered a bait are observed to determine if the bait is chewed to release vaccine in the oral cavity and any bait remnants or unconsumed baits are recollected. Generally, the same team of two people conducting OBH will concurrently perform DD vaccination to parenterally vaccinate dogs that can be held for vaccination. This combined approach can be termed OBH-DD.

### 2.4. Team Size

Each vaccination method requires a minimum number of people per vaccination team to achieve a given rate of vaccination. For example DD vaccination of dogs generally requires two staff; one vaccinator and one assistant or animal handler, whilst the CVR method often requires many more people; vaccinator, assistant, driver and net catchers (Box 1) [40]. The number of people per team may vary by setting depending on training, culture, expertise and the demography of the dog population. The number of people per team determines the total number of people required during the campaign.

There is an inverse relationship between campaign duration and workforce, as well as between the workforce availability and expertise. These factors must be offset to find a feasible solution to dog vaccination at scale. All of these factors are inter-related and must be considered when planning an effective mass dog vaccination campaign (Figure 1).

**Figure 2:**
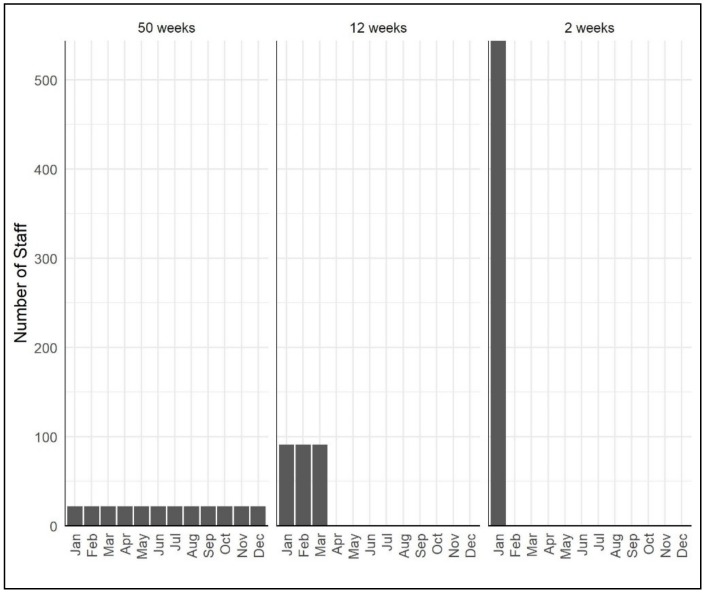
Staff requirement for campaigns vaccinating 70,000 dogs using CVR (11 dogs/person/day) and DD (41.5 dogs/person/day) methods over a period of 50 weeks, 12 weeks and 2 weeks requiring 22, 91 and 544 people respectively. Estimates of staff were generated using the VaxPLAN calculator (Supplementary Materials) [28].

As the campaign duration is shortened, the required number of teams, and therefore number of staff, increases (Figure 2, Supplementary Materials). With campaigns of less than two weeks, it is more likely that people will be able to participate either voluntarily or by way of diversion from other duties, therefore increasing the size of the potential workforce available [28]. Implementation of dog vaccination at national scale requires utilization of the government veterinary workforce and so identifying the campaign duration which makes this feasible is crucial to planning a national campaign structure.

As the duration of the campaign is extended, the number of vaccinations required per day reduces and so the number of teams, and therefore staff, can be reduced. The opportunity to utilise volunteers or temporary staff diverted from other roles is lessened unless they are rotated, in which case there is a continual need for training and supervision as new staff are brought up to speed. Therefore, the management requirement of this approach is greater and sustaining motivation and intensity of work becomes harder over time. Where the campaign cycle is less than 12 months, the months between campaigns can be a challenge because these staff must either be kept employed in other duties to avoid loss of expertise or must be re-recruited and trained each year. If reducing the duration of the campaign is considered prerequisite to gaining traction and sustaining the campaign, then overcoming aspects of training and human resource requirement must be prioritized.

Limitations to reducing a campaign period include the maximum available labour force, the ease of training staff in campaign methods and availability of fixed assets (i.e., vehicles, phones, cool boxes, equipment, etc.). As the size of a campaign increases, the availability of skilled workers may not fulfil the requirement, in which case the degree of training required to capacitate unskilled workers would increases. This does not present a problem where campaign tasks are routine to the workforce; however, where skills are specific to the campaign and not routinely taught at-large there will be a need for campaign-specific training which inevitably increases the complexity, cost and logistics of the initiative and may ultimately render the approach infeasible.

An example would be with the CVR method where skilled catchers work as a team using nets to catch inaccessible dogs for parenteral vaccination. Net catching requires weeks of training alongside experienced staff, physical agility, fitness and a good understanding of dog behaviour. Most regions do not have sufficiently large workforces routinely using nets to catch dogs who could be diverted to mass dog vaccination and therefore temporary staff would need to be trained in this method for a short-duration campaign. With more advanced methods of vaccination, it is often more cost-efficient and pragmatic to retain a permanent workforce of skilled staff year-round, than it is to repeatedly train a temporary team [24].

## 3. Historic Examples

### 3.1. Rabies Control in Latin America

The Latin American and Caribbean (LAC) campaigns are the largest example of sustained national dog rabies vaccination to date. LAC represents a human population of 641 million, spread throughout 42 independent countries [41]. The first mass vaccination campaign was conducted in Mexico City in 1969, followed by campaigns in several other Latin American capital cities through the 1970s [42,43]. A formalized regional elimination effort was conceived in 1983, with the Pan American Health Organisation (PAHO) coordinating the initiative initially in urban centers, which expanded into rural regions from 1991 [44]. The campaign was synchronized over a period of no more than one week every year, vaccinating over 50 million dogs annually at its peak in 2009 [45,46].

This rapid expansion of vaccination activities in LAC countries is likely to have been partly possible due to the progressive growth of infrastructure and prosperity, resulting in changes in dog ownership practices and capacity to orchestrate large scale campaigns [29]. The UN’s human development index (HDI) is a useful measure of development for considering a nations intrinsic capacity for large scale public health campaign implementation and may be of use when comparing regions for potential campaign scale-up [29]. The mean Human Development Index of LAC countries increased from 0.61 in 1990 to 0.71 in 2009 [47], which compares with the HDI of India in 2017 of 0.64.

The priority to first establish an effective approach in urban centres had a number of operational advantages. Urban settings represent the greatest at-risk human populations as well as generally having the highest dog population densities [42] and therefore successful public health campaigns in such areas are often a political priority for local Governments [48,49]. Focal studies in Africa have demonstrated that the large dog population creates a high workload in urban centres; however, the greater availability of infrastructure and workforce makes high output interventions more feasible in the shorter term and generally present as a reduced cost per vaccine dose delivered, comparative to campaigns in lower density rural regions [21,50,51,52,53].

Unfortunately, human rabies deaths often occur in marginalised communities and rural areas where access to healthcare and post exposure prophylaxis is more limited than in urban settings [54,55,56]. Additionally there is evidence from African settings that rabies circulates in the peri-urban regions and is regularly re-introduced into cities [20,57]. Therefore to demonstrate a truly effective campaign, it is important that the successes gained in urban centres are then expanded into peri-urban and rural areas as quickly as possible, as was conducted in LAC countries by the 1990s [44].

The rapid planning and development of large urban campaigns creates the opportunity to recruit and establish local leadership and technical capacity for mass dog vaccination. The knowledge, expertise and political momentum gained in these urban campaigns is then able to support the development of wider reaching rural campaigns with leaders lobbying government to sustain and expand the elimination effort as a ‘ripple effect’ across a region [58].

The short campaign duration of less than one week enabled coordination of activities across the subcontinent, generating huge awareness through mass media and engaging with every sector of society [59]. The scaling and success of such an approach was aided by the fact that a high proportion of the roaming dog population were owned and accessible throughout most Latin American communities [44,60,61,62,63,64]. This meant that when free dog vaccination was made available at central point locations, the desired herd immunity was achieved through motivated and mobile dog owners taking their dogs for vaccination. It is possible to run a vaccination clinic through the central point method with minimal additional training and expanding the approach was possible through broad recruitment of the public and private veterinary workforces. This was achieved by condensing the campaign duration into a short period of time, making participation across government, non-governmental organisation (NGO) and voluntary sectors possible.

The overarching coordination provided by PAHO was critical to supporting and sustaining the regional rabies elimination effort [42,44]. PAHO not only provided consultancy on campaign evaluation and training, but also a central mechanism for obtaining permanent funding to sustain priority campaigns through the revolving fund [43]. The LAC rabies elimination campaign is now in its last mile, with the disease persisting in pockets where additional focus and innovation is now required to eliminate the disease [39].

It took over 20 years for dog vaccination activities to mature from focal efforts in urban settlements to widespread, centrally funded activities across both urban and rural areas. Although dog accessibility is favourable in comparison to many other endemic regions, concentration of the campaign into a 1 week period maximised community engagement and contribution. Finally central coordination and permanent funding allowed for a long term strategy to be enacted, regardless of local national priorities.

### 3.2. Transferrable Lessons from Polio Elimination in India

The Global Polio Eradication Initiative launched in 1988 and has now eliminated wild poliovirus from all but three countries [65] and represents the largest international health initiative in history. There are many operational lessons to take from the initial progress towards global momentum, scaling of activities and the final struggles to achieve elimination [66].

International coordination of vaccination activities was crucial in making strides towards regional polio elimination. This coordination was made possible due to agreement between countries to synchronize massive vaccination efforts on a single day, therefore mobilizing enormous resource across large parts of the globe [67,68]. Synchronization of campaigns to vaccinate every child under the age of 5 years in numerous Asian countries in the late 1990s enabled the vaccination of 248 million children during two days in 1997 and 1998 [69]. Efforts were intensified in high-risk areas or where coverage was lower through the use of subnational immunization days and house-to-house ‘mop-up’ campaigns [70,71]. The ability to synchronize massive vaccination pulses across countries and continents enabled rapid progress towards elimination in operationally favourable regions, which were then followed by more refined, targeted areas in regions where penetration was lacking. A similar view point may be helpful as policy makers look to scale dog vaccination efforts; however, a method of vaccination which can be deployed across vast geographic areas over a short period of time is prerequisite to this approach.

The simplicity of oral polio vaccine administration, applying a drop of vaccine to the tongue, meant that health workers required minimal additional training in vaccine administration and, in cases where parents were supportive of the campaign, accessing children was relatively straight forward. In the case of dog vaccination, training in parenteral vaccine administration and dog handling are additional operational barriers to mass deployment. Developing a latent workforce with the ability to vaccinate dogs, whilst maximising the basal competence of communities to restrain owned/community dogs are areas which still require further attention in rabies control.

Social mobilization was reportedly crucial to the successful acceptance and penetration of polio immunization to every area of every country across the globe [70]. The widespread engagement of all segments of society included policymakers, opinion leaders, the media, technical experts, religious groups, the private sector, NGOs and community members [70]. As polio vaccination campaigns scaled-up, the focus around vaccinating ‘every child under the age of 5′ made the objective of the campaign clear. A similar scale of community engagement would need to be achieved in national mass dog vaccination activities, with a comparable clarity around the expectation that every owned dog should be rabies vaccinated. This may help to place an emphasis on dog owners to be able to hold and present their dog for vaccination as a minimum cultural requirement.

The use of ‘micro-plans’ were reported as crucial to the success of many large scale human vaccination initiatives, including those for control of polio, measles-rubella and cholera [72,73,74,75]. Mobile technology has already been demonstrated for remote direction of dog vaccination teams to specific geographic regions through Google Maps, which would support the implementation of such micro-plans for rabies control [76].

Although beyond the scope of this review, it is important to note that the success of the polio elimination campaign has also depended on the creation of an effective and robust international surveillance network and diagnostic infrastructure to monitor impact and tailor vaccination activities according to polio incidence [77].

## 4. Indian Metropolis Projection

**Figure 3:**
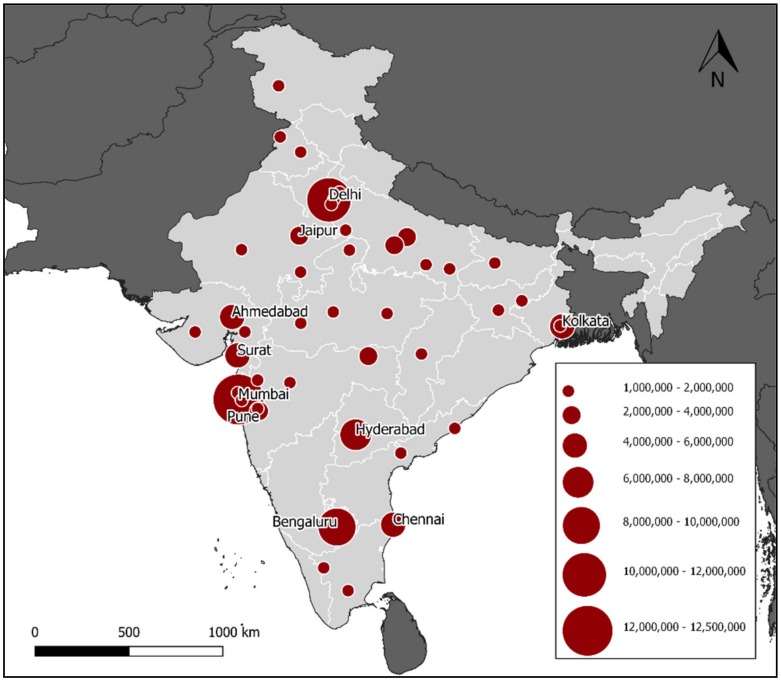
Location of the 46 cities in India with a population of over 1 million people (2011 census), illustrating the need for dog vaccination campaign structures which can be implemented on a massive scale across numerous metropolis settings. The top 10 populous cities are labelled by name.

India has a huge logistical and practical challenge ahead in the control of canine rabies. Large free roaming dog populations, areas where inaccessible dogs predominate and challenging terrain will hamper the efficient delivery of canine vaccination nationally. According to the 2011 national census, there are 46 cities with a population of over one million people (Figure 3), which collectively accounted for approximately 10% of India’s population at that time [78]. As was seen in the Latin America Campaign, the process of developing solutions for these urban regions will generate experience and understanding of methods that would support the efficient expansion into other areas. Therefore, data were reviewed from past campaigns in India and dog population estimates to project hypothetical campaign structures for a municipality-wide campaign in Bangalore City, Karnataka, India to review the feasibility of available methods.

Although both of the above examples used a predominantly central point approach to vaccination, this is unlikely to achieve the required rates for vaccination of dogs in India due to the large roaming and loosely owned dog population. Therefore, combinations of other approaches have been considered.

## 5. Estimated Dog Population Size

Reliable estimates for roaming dog population size in Indian cities are limited. There is wide variation in reported estimations for human-to-dog ratios in urban settings of India, ranging from 23:1 to 83:1 (Table 1), with a mean of 50:1 (Table 1) [19,79,80,81,82,83,84,85]. Estimations from urban regions of neighbouring countries also vary greatly, with an estimated human-to-dog ratio of 828:1 in Dhaka, Bangladesh [19], and 16:1 in urban areas of Bhutan [84]. More data is needed to accurately estimate the human-to-dog ratio for different settings, accounting for factors including pet ownership practices, religion, culture, human population density and geography. Although having an accurate estimate of population size can be helpful in planning pilot activities, it should not prohibit or delay their implementation and dog population estimates should not be used as the basis to estimate vaccination coverage which requires direct evaluation through post-vaccination surveys [86,87]. The value of well-planned and coordinated pilot campaigns that engage local stakeholders cannot be underestimated in their potential to catalyse the further expansion of activities. Such pilot initiatives, when coupled with post-vaccination surveys, not only provide an estimated dog population size, but also generate experience in delivering canine vaccination within the community, understanding of dog accessibility and give operational insights into the cost and efficacy of trialled methods [18,21]. These foundations enable adaptation of methods, systems, tools and training to meet local needs, as well as confidence to both campaign planners and funders in reaching for larger scale initiatives.

In order to evaluate the feasibility of different approaches, despite the lack of available data for the size of the dog population, three scenarios were projected for estimating the dog population of Bangalore city; (A) best case with a human-to-dog ratio of 83:1, (B) a middle estimate based on the mean human-to-dog ratio from reported urban settings in India of 50:1 and (C) worst case scenario of a human-dog-ratio of 23:1.

## 6. Vaccination Team Size

Methods that have been described for mass dog vaccination in India include combinations of DD (teams of two), CVR (teams of seven), and DD-OBH (teams of two) [18,40]. Box 1 describes the various vaccination methods and their combinations. In Goa, the CVR and DD methods are conducted by different teams to vaccinate inaccessible and accessible dogs respectively, whereas where OBH is used, it is combined with the DD method so that the same team do both approaches. CP vaccination has not been considered due to the lack of documented examples of its use for mass dog vaccination in India.

## 7. Rate of Vaccination

There are limited reported examples of the rate of vaccination using different methods in Indian settings. For the purpose of estimating campaign resource requirements, a parallel CVR and DD approach (using separate teams) was compared with a combined OBH-DD approach (using teams conducting both methods) (Box 1). Details for how the rate of vaccination for each method was estimated are outlined below and are summarized in Table 2.

### 7.1. CVR Rate of Vaccination

**Figure 4:**
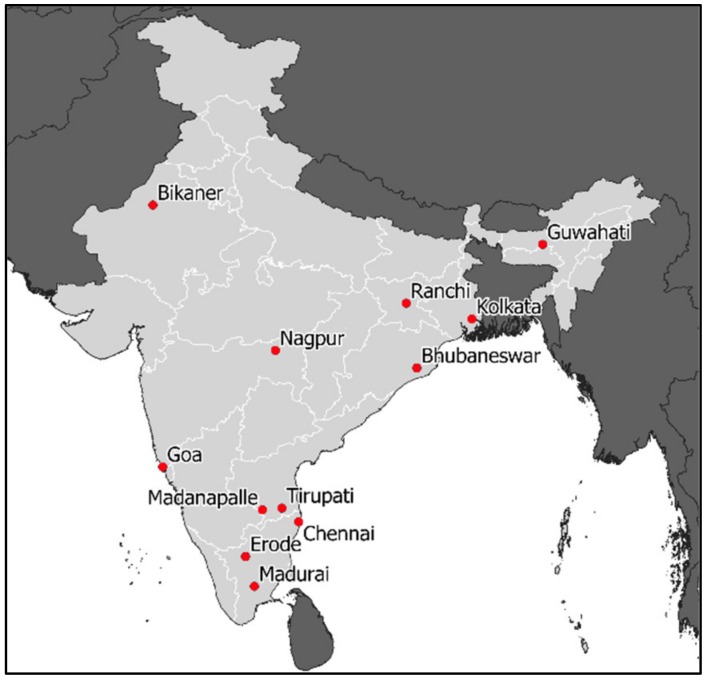
Location of synchronized pilot campaigns in September 2013.

**Figure 5:**
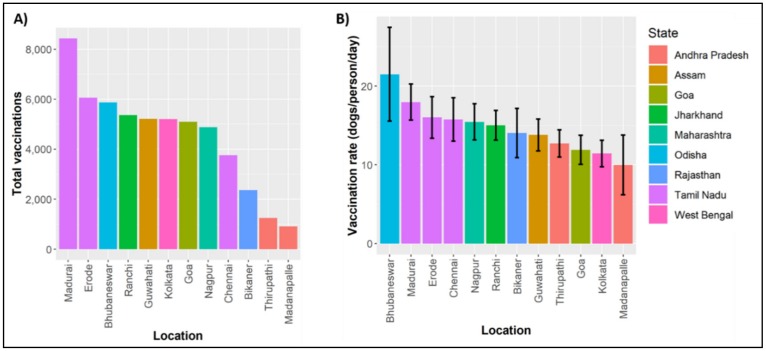
Charts of (**A**) the total number of dogs vaccinated by region and (**B**) the mean rate of vaccination per person per day.

During September 2013 (01/09/2013–30/09/2013), Mission Rabies conducted synchronized pilot mass dog vaccination campaigns in 12 urban locations using the CVR vaccination approach (Figure 4). The details of 54,227 dogs vaccinated during the campaign were recorded electronically using a smartphone application, including the date, time, user, age, sex and health of the dog [76]. The mean rate of vaccination across project sites was 102 dogs/team/day (95% CI: 72–139), which represents 14.6 dogs/person/day (CI: 10–20) (Figure 5). Post vaccination evaluations were conducted at five project sites, estimating mean coverage of 71.2%.

### 7.2. DD Rate of Vaccination

Data from the 2018 and 2019 Goa vaccination campaign were used to estimate the rate of vaccination for DD teams, which were working in parallel with separate CVR teams focusing on inaccessible dogs. Data from the Goa vaccination campaign came from DD teams vaccinating in regions with high human density (more than 1500 people/km^2^). Between 01/10/2018 and 31/07/2019, DD teams vaccinated 8266 dogs over 109 days in regions with human density of over 1500 people/km^2^. The mean rate of vaccination was 47.8 dogs per day (23.9 dogs/person/day).

### 7.3. OBH-DD Rate of Vaccination

A recent study compared the CVR method with an OBH approach across land types in Goa State, India [40]. The urban rate of vaccination with OBH-DD was 83 dogs/team/day (41 dogs/person/day). The mean human density of ‘urban’ areas in this study was approximately 1494 people/km^2^, which is considerably lower than that of Bangalore. Therefore, it is likely that the rate of DD vaccination would also be higher in Bangalore city.

## 8. Campaign Duration

A maximum campaign duration of 2 weeks was considered feasible for the participation of the government veterinary work force as well as local NGOs, volunteers and private veterinarians. This would require the target dog population to be vaccinated within 11 working days (based on a 5.5 day working week).

## 9. Vaccination Team Direction

**Figure 6:**
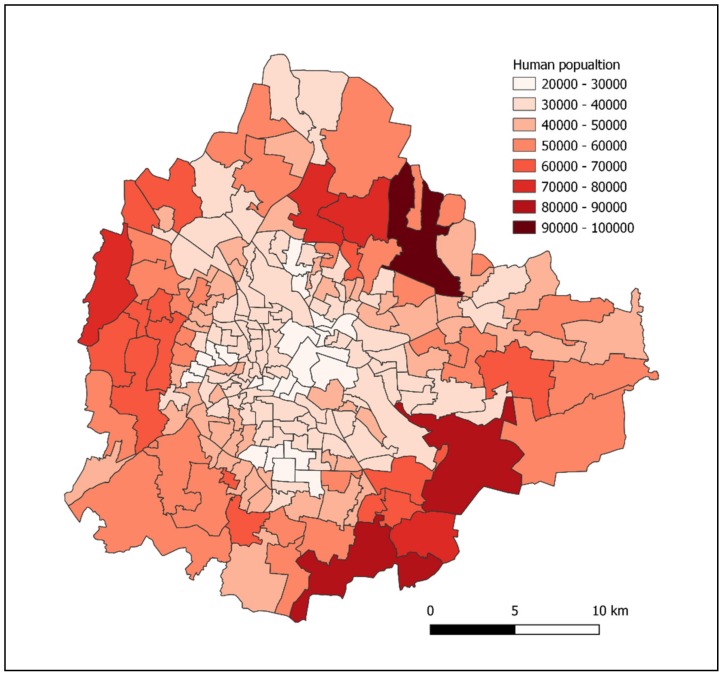
Map showing human population by ward (India census 2011), which would be the basis for team direction using campaign management technology, such as WVS App, although further division into smaller Working Zones denoting a team’s area of work for a few days would likely be needed.

To achieve homologous coverage and avoid pockets where rabies virus could potentially persist within the population, teams would need to be coordinated across the municipality to systematically reach every ward and block. The use of mobile technology to manage large numbers of teams in near real-time has been demonstrated for this purpose at scale [76]. Human census data by municipal ward can give an indication of likely resource requirement at a higher resolution (Figure 6); however, these regions would need to be further divided into working zones, which could be covered by a vaccination team within a day or two.

## 10. Estimate of Campaign Resource Requirement

The best case scenario for Bangalore city estimated a dog population of 102,000 dogs, with a middle estimate of 167,000 dogs and a worst case scenario of 367,000. The VaxPLAN tool was used to estimate the resource requirement, vaccination coverage and operational cost of the three scenarios (Table 3, Supplementary Materials) [28].

The number of vaccination staff that would be required in the best case scenario using the CVR-DD method for vaccination of inaccessible dogs was 448 as compared to 211 using the OBH approach. Even at this optimistic scale, the need for 64 vehicles to transport net catching teams and the training required to train 256 net catchers (four per team) would be logistically challenging. Even if the team structure were adjusted to three catchers per team, this would still require 192 competent net catchers for two weeks, which is unlikely to be achievable or manageable. This compares with 106 OBH teams moving on scooters vaccinating accessible dogs parenterally and inaccessible dogs with an oral rabies vaccine (ORV).

Even if ORV were not available, these DD teams could focus only on the owned and friendly roaming dogs in the first instance. This would engage with the dog owning public and encourage a culture where people are expected to present their dogs for vaccination when a vaccination team visits their house. Should ORV be made available at a later time, the experience, expertise and capability to conduct DD parenteral vaccination would have already been established and would therefore provide a strong foundation on which to expand capacity using ORV.

As the estimated dog population increases through Scenario B and C, the feasibility of the CVR approach diminishes, with 739 and 1604 staff needed respectively. Even at these larger scales, the OBH approach remains potentially feasible, requiring 316 staff travelling on 158 scooters in the middle scenario. In the worst case scenario of 250,000 dog vaccinations in two weeks, an estimated 684 staff would be required for the OBH-DD approach, which would be a challenging prospect to manage and train. At higher dog population estimates, the number of staff required can be reduced by extending the vaccination period; however, this results in the problems described in the previous section.

The estimated vaccination coverage and cost of the two approaches was comparable between the different methods (Table 3).

## 11. Discussion

This review highlighted the importance of short-duration vaccination campaigns in enabling the continental mobilization of vaccination workforces in both the Latin America rabies control effort and the global polio eradication campaign. The extrapolation of human resource requirements using existing dog vaccination methods in India revealed that short-duration campaigns are unlikely to be feasible. To benefit from the operational advantages of short-duration pulse vaccination campaign structures, novel vaccination approaches should be explored.

India’s HDI of 0.64 in 2017 was comparable to that of Latin America at the time of expanding mass dog vaccination activities in 1990 (0.61), indicating that India may already have the monetary and infrastructural capacities to facilitate successful intensive dog vaccination campaigns. In contrast to Latin America, however, the large proportion of roaming dogs in India that cannot be readily captured for parenteral vaccination presents considerable barriers to rapid campaign expansion. Both the polio eradication campaign and dog vaccination in Latin America leveraged community participation to access the target population for vaccination through the presentation of children and owned dogs to vaccination teams respectively. Where the target population is less readily accessible, more intensive methods must be employed to achieve herd immunity, thereby increasing campaign complexity.

This study found that the large number of specialised vaccinators required for the vaccination of inaccessible dogs through the CVR method would likely prohibit its use in short-duration dog vaccination campaigns at scale. To achieve successful, large-scale, short-duration vaccination campaigns, more efficient vaccination methods that do not rely on specialised vaccinators are needed for the vaccination of inaccessible dogs. The use of oral rabies vaccination of dogs has recently been described as a possible solution to mass dog vaccination at scale due to the ability to vaccinate high numbers of parenterally-inaccessible dogs per staff member (i.e., increased vaccination efficiency among the target population) [40]. Two-person vaccination teams can be quickly trained in the OBH method to vaccinate inaccessible dogs, whilst concurrently vaccinating accessible dogs parenterally when they are presented to the OBH teams by their caretakers [40]. This approach has been demonstrated to be safe, efficient and effective for the vaccination of dogs [40,88,89]. Despite the higher cost of ORV compared to parenteral vaccine, the VaxPLAN calculator estimated total campaign cost for the CVR-DD and OBH-DD methods to be similar, although the personnel needs of OBH-DD were far more realistic. The necessary permissions for the use of ORV under field conditions must be secured to further evaluate the OBH method in India.

A graduated approach to scaling mass dog vaccination efforts whilst varying implementation by land type, population density, culture and geography would be sensible. Successful approaches in metropolitan areas will not necessarily be successful across large rural areas. It is only by creating a conversation around these concepts that it is possible to distil what the approach to rabies control might look like across the diverse cultural and geographic landscapes of India. Openly available tools help to rapidly forecast campaign requirements based on preliminary pilot data [28], developing early examples of success upon which to expand activities more broadly as was effective in the progressive growth of the Latin American dog vaccination campaigns through the 1970s and 1980s. The messaging around polio immunization was clear; every child under the age of 5 years must be vaccinated. In the context of rabies control, the objective of vaccinating every dog in India is not feasible due to the large stray population, and, consequently, would be unlikely to instil confidence in staff or the public in the potential to achieve rabies elimination. That said, the campaign messaging needs to foster the same social mobilization as that achieved by the polio campaign. A broad objective to vaccinate over ‘70% of the dog population’ is not clearly actionable by vaccination staff in the field and does not provide clear direction to the dog owning public as to what is required from them. A campaign message to vaccinate ‘every dog, every year’ as the priority, would provide a strong starting point on which to build methods to then access the unowned population.

Widespread community engagement and awareness as well as robust case reporting, disease surveillance and response are crucial to the success of elimination efforts and these aspects should be developed in parallel as programs grow. It is important to ensure that the public are properly educated both on what is happening during the campaigns, to avoid fear of dogs, and on how best to live with dogs to reduce the number of bites [90]. The use of smartphone applications to direct and evaluate emerging strategies will be of enhanced benefit as a methodology for national canine rabies elimination is sought [76]. If OBH is to be considered, early stages of implementation would greatly benefit from the use of smartphones to track efficiency and effectiveness, in addition to centralised reporting of any human vaccine contact events as required by WHO [11]. 

In the same way that the ambition to eliminate poliomyelitis had a multitude of secondary benefits in accelerating the development of healthcare infrastructure and disease surveillance systems in developing countries, momentum to eliminate rabies would also leave the legacy of enhancing collaboration between human and animal health services and systems to help address other One Health issues [66,91]. 

## 12. Conclusions

Mass dog rabies vaccination remains in its infancy in many rabies endemic areas of the world and a workable solution at a national scale is yet to be identified and trialled in India. Historic examples of continental vaccination efforts used short-duration campaigns to galvanize public awareness and make use of massive temporary vaccination workforces from government health sectors and non-governmental organisations; however, accessible target populations were critical to success. If India is to rely upon parenteral vaccines for canine rabies elimination, the high proportion of inaccessible roaming dogs will necessitate more complex vaccination methods, such as CVR, which require sizeable numbers of skilled people, thus prohibiting large-scale short-duration campaigns. More efficient, low-skill techniques to immunize inaccessible dogs using oral rabies vaccine alongside parenteral vaccination of accessible dogs, will allow the campaign duration to be reduced and facilitate implementation at larger scale.

The outcome of this review found that implementing a vaccination strategy using the core concepts of India Polio eradication or Latin America rabies elimination would require the large-scale availability of oral rabies vaccines for dogs. Further exploration of the use of oral rabies vaccination as a complementary tool to scale up short-duration campaigns in India is urgency needed.

## Figures and Tables

**Table 1:** Table of available literature for roaming dog population estimates of urban settings in India as well as a study from Bangladesh and Bhutan. Rank refers to ranked order of the city within all Indian cities according to the 2011 India human census. Human and estimated canine populations refer to those reported in the source article. Where human population is not given in the article, an estimate from the time of the study was used.

Rank	State	City	Human Population	Estimated Dog Population	H:D Ratio	Source
3	Karnataka	Bangalore	8,436,675	200,000	42.2	Sudarshan et al. (2001)
10	Rajasthan	Jaipur	3,046,163	36,580	83.3	Hiby et al. (2011)
20	Gujarat	Vadodara	1,818,925	44,018	41.3	Kartal et al. (2017)
41	Tamil Nadu	Coimbatore	1,890,000	46,292	40.8	Kartal et al. (2018)
43	Rajasthan	Jodhpur	1,033,918	24,853	41.6	Hiby et al. (2011)
86	Gujarat	Jamnagar	609,613	25,768	23.7	Kartal et al. (2017)
NA	Bangladesh	Dhaka	15,391,000	18585	828	Tenzin et al. (2015)
NA	Bhutan	Multiple	NA	NA	16.3	Rinzin et al. (2016)

**Table 2:** Calculations for estimating team and per person rate of vaccination for different methods.

Method	OBH-DD	CVR	DD
Source	Gibson et al. (2019)	MR launch data	Goa vaccination campaign
DD team rate (dogs/team/day)	19	0	47.8
OBH/CVR team rate (dogs/team/day)	64	102	0
Overall team rate (dogs/team/day)	83	102	47.8
Number of staff per team	2	7	2
DD staff rate (dogs/person/day)	9	0	23.9
Alternative method staff rate (dogs/person/day)	32	14.6	0
Overall staff rate (dogs/person/day)	41	14.6	23.9

**Table 3:** Table showing estimations for staff and teams required to vaccinate 70% of the dog population in Bangalore City over a two week period using either catch-vaccinate-release (CVR) or oral bait handout methods (OBH) based on three scenarios for potential dog population size. Scenarios for dog population size in Bangalore city were calculated based on best, mean and worse case scenarios for available dog-to-human ratios in Indian urban settings; (A) 83:1, (B) 50:1, (C) 23:1.

	Scenario A		Scenario B		Scenario C	
**Duration**	2 weeks		2 weeks		2 weeks	
**Human population**	8,436,675		8,436,675		8,436,675	
**Dog:human ratio**	83		50		23	
**Estimated dog population**	101,647		168,734		366,812	
**Target coverage (%)**	70		70		70	
**Total campaign required vaccinations**	71,153		118,113		256,768	
**Number of working days**	11		11		11	
**Vaccination method**	CVR-DD	OBH-DD	CVR-DD	OBH-DD	CVR-DD	OBH-DD
**DD staff estimate**	84	211	126	316	272	684
**CVR staff estimate**	364	0	613	0	1332	0
**Total staff estimate**	448	211	739	316	1604	684
**Overall vaccination coverage estimate**	72%	79%	71%	71%	71%	71%
**Roaming dog coverage estimate**	66%	75%	66%	67%	66%	66%
**Cost per vaccine delivered (USD)**	2.53	2.57	2.33	2.45	2.19	2.31
**Annual campaign cost (USD)**	184,956	205,903	278,783	293,655	568,206	599,315

## Supplementary Materials

The following are available online at https://www.mdpi.com/2414-6366/5/1/47/s1.
